# Long-Term Association of an Intensive Lifestyle Intervention on Mortality: The Look AHEAD Study

**DOI:** 10.1093/geroni/igaf122.469

**Published:** 2025-12-31

**Authors:** Denise Houston, Joni Evans, Charles Semelka, Haiying Chen, Peter Huckfeldt, Marcel Salive, Mark Espeland, Lynne Wagenknecht

**Affiliations:** Wake Forest University School of Medicine, Winston-Salem, North Carolina, United States; Wake Forest University School of Medicine, Winston-Salem, North Carolina, United States; Wake Forest University School of Medicine, Winston-Salem, North Carolina, United States; Wake Forest University School of Medicine, Winston-Salem, North Carolina, United States; University of Minnesota, Minneapolis, Minnesota, United States; National Institutes of Health, Rockville, Maryland, United States; Wake Forest University School of Medicine, Winston-Salem, North Carolina, United States; Wake Forest University School of Medicine, Winston-Salem, North Carolina, United States

## Abstract

Although weight loss is recommended for individuals with type 2 diabetes and obesity to improve diabetes- and obesity-related risk factors, few studies have examined the effects of intentional weight loss on mortality. Look AHEAD, a randomized trial in 5,145 individuals aged 45-76 yrs with type 2 diabetes and overweight/obesity comparing an intensive lifestyle intervention (ILI) designed to achieve 7% weight loss to diabetes support and education (DSE), previously found no significant differences in all-cause mortality during 10 yrs of intervention or at 17 yrs of follow-up. Since the effects of intentional weight loss on mortality risk may take many years to emerge, we conducted a follow-up examination of all-cause mortality over 23 yrs of follow-up using proportional hazards regression. Interactions between ILI and mortality were also examined in pre-specified subgroups. A total of 800 deaths in ILI and 861 deaths in DSE were observed over 80,725 person-yrs. ILI experienced a lower incidence of all-cause mortality relative to DSE (HR [95% CI]: 0.89 [0.81, 0.98]). There were no significant interactions between treatment and age, sex, or cardiovascular disease history. However, there were significant race/ethnicity by treatment interactions (p = 0.01), with HRs (95% CIs) for participants identifying as African American 0.96 (0.75, 1.23), Caucasian 0.94 (0.84, 1.05), and Hispanic 0.54 (0.39, 0.74). In middle aged and older adults with type 2 diabetes and overweight/obesity, intentional weight loss modestly reduced all-cause mortality risk over 23 yrs of follow-up, which appeared to be largely driven by a reduction in mortality among participants of Hispanic ethnicity.

